# Multiple Myeloma Uncovered Under Excessive Antacid and Chronic NSAID Use in a Young Female

**DOI:** 10.7759/cureus.27629

**Published:** 2022-08-03

**Authors:** Priscilla Fujikawa, Kenneth Brand, Siddharth Shah, Viraj Munshi, Kashyap Patel

**Affiliations:** 1 Internal Medicine, LewisGale Medical Center, Salem, USA; 2 Internal Medicine, Edward Via College of Osteopathic Medicine, Blacksburg, USA

**Keywords:** plasma cell dyscrasia, vrd, hypercalcemia, crab, multiple myeloma

## Abstract

Multiple myeloma (MM) is a plasma cell dyscrasia in which nearly all cases are diagnosed in patients over 40 years old. This report illustrates a case of a young female who presented with severe generalized weakness, acute kidney injury, hypercalcemia, and anemia. Her symptoms were initially attributed to chronic NSAID and antacid intake, especially given her young age. However, further workup was pursued to rule out other potential diagnoses despite her age. She was ultimately diagnosed with multiple myeloma and started on bortezomib, cyclophosphamide, and dexamethasone. This report emphasizes the importance of maintaining a broad differential diagnosis. Untrained physicians can easily overlook rare cases. Timely diagnosis and treatment are key, and therefore, a high degree of suspicion is crucial for this patient population.

## Introduction

Multiple myeloma (MM) is a plasma cell dyscrasia that accounts for almost 1% of new neoplasms. Incidence increases with age, and the risk of developing MM is highest in those older than 65 years of age. Of patients diagnosed with MM, 99% are 40 years and older. The incidence of death from multiple myeloma has doubled in the past 30 years [1]. Our case is interesting in that it falls under 1% of cases diagnosed under 40 years old. The patient in this report was 39 years old and had several plausible explanations for her symptoms. Her epigastric pain and acute kidney injury were initially attributed to chronic NSAID use. Her hypercalcemia was initially attributed to excessive antacid intake. We here present a unique case of multiple myeloma in a young female.

## Case presentation

The patient was a 39-year-old African American female with a history of asthma who presented with one month of epigastric pain, vomiting, and generalized weakness. The epigastric pain was sharp, radiated to the bilateral lower chest, and was worse with movement and deep breaths. She also had constant and sharp low back pain that prevented her from ambulating independently. The pain radiated to the posterior and lateral aspects of her right leg for two months in the same region where she had previously had trauma as a child. She had been taking over-the-counter NSAIDs and antacids multiple times a day for several months (unable to quantify exactly how many) to help control her pain. Prior to this, she was healthy, independent of her activities of daily living, and worked as a teacher. She had never had an esophagogastroduodenoscopy or colonoscopy. On admission, her hemoglobin was low, while creatinine and calcium were elevated (Table 1).

**Table 1:** Laboratory values Hemoglobin, creatinine, and calcium levels on admission and three months prior

Laboratory value	On admission	Three months prior (outpatient)	Reference range
Hemoglobin (g/dL)	9.8	11.8	11.4-15.5
Creatinine (mg/dL)	15	1.16	0.6-1.3
Calcium (mg/dL)	15	11	8.7-10.2

A lumbar spine X-ray two months prior showed L4-L5 disc degeneration, but no acute pathology. The chest X-ray did not show hilar adenopathy or other pathology. Initially, her acute kidney injury was thought to be secondary to chronic NSAID use, and her hypercalcemia was attributed to excessive antacid intake. However, further workup was pursued to rule out other uncommon diagnoses at her age. This then revealed a free kappa to lambda light chain ratio of 1,935 (reference: 0.26-1.65) and a random urine protein electrophoresis M-spike of 42.5. The bone marrow biopsy contained >50% plasma cells, and flow cytometry showed kappa-restricted plasma cells that expressed CD38 and CD138 but were negative for CD117 and CD56. Fluorescence in situ hybridization showed deletion of 13q and loss of 4p. She was diagnosed with multiple myeloma and was started on bortezomib, cyclophosphamide, dexamethasone, and acyclovir. After the initiation of dialysis, her creatinine decreased to 3.6 mg/dL, and her ionized calcium normalized to 8.9 mg/dL. She was ultimately discharged to inpatient rehabilitation.

## Discussion

MM involves either chromosomal aneuploidy or IgH locus translocations (most frequently involving cyclin D dysregulation), resulting in the development of monoclonal gammopathy of unknown significance (MGUS). The accumulation of genetic aberrations, including the deletion of 13q, amplification of 1p, and deletion of 1p, results in the progression to smoldering multiple myeloma (SMM). The progression of SMM to MM involves a secondary genetic event, including light chain translocations, RAS gene mutations, NF-kB pathway activations, and 17p deletions [2]. The transition between MGUS, SMM, and MM (both intra- and extra-medullary) depends on reciprocal interactions contained in the bone marrow microenvironment, which are mediated by various cytokines (IL-6 and IGF1), adhesion molecules (MMSET), and cellular receptors (FGFR3) [3,4].

The acronym CRAB is often used in the diagnosis of multiple myeloma (MM): hyperCalcemia, Renal insufficiency, Anemia, and Bone disease [5]. Our patient had three of those four features, which raised our suspicion about MM despite her young age. The pathogenesis of renal failure is multifaceted and involves free light chains and hypercalcemia [6,7]. Light chain-mediated renal injury involves the aggregation of light chains with Tamm-Horsfall proteins into myeloma casts, leading to tubular obstruction, inflammation, and fibrosis. The deposition of light chains in the glomerulus can lead to amyloid glomerulonephritis/primary amyloidosis [7]. Normocytic, normochromic anemia is present in about 70% of patients at diagnosis [5]. This stems from erythropoietin deficiency in the setting of renal disease and bone marrow infiltration [8]. Bone marrow infiltration and hypogammaglobulinemia due to B-cell insufficiency increase the risk of infections, which is the leading cause of mortality in this population. One of the most common infections is the herpes zoster virus [9]. That guided our decision to initiate acyclovir prophylactically in our patient.

The diagnosis of MM can be made if there are ≥10% clonal plasma cells in the bone marrow or soft tissue/bone plasmacytoma and damage to one of the organs in the acronym CRAB or evidence of end-organ damage (60% or more plasma cells in the bone marrow, involved-to-uninvolved free light chain ratio of 100 or more, and more than one bone or bone marrow lesion on MRI or PET scan) [10,11].

Myeloma therapy is typically divided into induction and maintenance stages with differentiation based on whether or not a patient is a transplant candidate. Before undergoing therapy, care must be taken with respect to the patient’s expected outcome, performance status, clinical presentation, and the availability of different therapies [12].

Most transplant-ineligible patients can undergo the same regimens as those who are transplant-eligible, with some exceptions [12,13]. Although three-drug therapies (bortezomib, cyclophosphamide, dexamethasone or bortezomib, lenalidomide, and dexamethasone) are preferred, two-drug regimens (lenalidomide and low-dose dexamethasone) can be considered in the case of elderly or frail patients [14]. In these cases, dose-adjusted bortezomib, lenalidomide, and low-dose dexamethasone (termed VRD-lite) can have comparable efficacy and increased tolerability to the standard regimen [5].

Hematopoietic cell transplant (HCT)-ineligible patients receive 8-12 cycles of induction therapy before progressing to maintenance therapy, which typically consists of lenalidomide or bortezomib monotherapy [15]. The latter can be a reasonable choice if bortezomib-based therapy was also used for primary treatment [11].

Since the goal of the autologous transplant is to replenish the marrow with healthy cells, a 3- to 4-month duration of induction therapy is performed prior to stem cell collection, with hopes of minimizing the deleterious cells transplanted. HCT-eligible patients undergo a similar induction therapy, as three-drug regimens are the standard of care for primary treatment in all HCT-eligible patients [15].

For both transplant and non-transplant candidates, typically, VRD (bortezomib, lenalidomide, and dexamethasone) therapy is appropriate as induction therapy [15]. In this case, our patient received cyclophosphamide rather than lenalidomide due to her renal failure.

## Conclusions

This case highlights the key to being a successful internist: keeping a broad differential diagnosis. Although the initial presenting signs and symptoms could have been explained by chronic NSAID use and excessive antacid intake, an accurate diagnosis and timely initiation of treatment were only possible when the decision was made to investigate other rare diseases for her age.
